# Correlation between anti-hypertensive drugs and disease progression among moderate, severe, and critically ill COVID-19 patients in the second referral hospital in Surabaya: A retrospective cohort study

**DOI:** 10.12688/f1000research.51785.2

**Published:** 2021-09-24

**Authors:** Satriyo Dwi Suryantoro, Mochammad Thaha, Mutiara Rizky Hayati, Mochammad Yusuf, Budi Susetyo Pikir, Hendri Susilo

**Affiliations:** 1 Department of Internal Medicine, Faculty of Medicine, Universitas Airlangga, Surabaya, East Java, 60132, Indonesia; 2 Universitas Airlangga Hospital, Surabaya, East Java, 60115, Indonesia; 3 Department of Cardiology and Vascular Medicine, Faculty of Medicine, Universitas Airlangga, Surabaya, East Java, 60132, Indonesia

**Keywords:** COVID-19, Severity, Progression, Hypertension, Anti-hypertensive drugs, Infectious disease

## Abstract

**Background**: Hypertension, as the comorbidity accompanying COVID-19, is related to angiotensin-converting enzyme 2 receptor (ACE-2R) and endothelial dysregulation which have an important role in blood pressure regulation. Other anti-hypertensive agents are believed to trigger the hyperinflammation process. We aimed to figure out the association between the use of anti-hypertensive drugs and the disease progression of COVID-19 patients.

**Methods**: This study is an observational cohort study among COVID-19 adult patients from moderate to critically ill admitted to Universitas Airlangga Hospital (UAH) Surabaya with history of hypertension and receiving anti-hypertensive drugs.

**Results**: Patients receiving beta blockers only had a longer length of stay than angiotensin-converting enzyme inhibitors (ACEIs) and angiotensin receptor blockers (ACEI/ARB) or calcium channel blockers alone (17, 13.36, and 13.73 respectively), had the higher rate of intensive care unit (ICU) admission than ACEi/ARB (p 0.04), and had the highest mortality rate (54.55%). There were no significant differences in length of stay, ICU admission, mortality rate, and days of death among the single, double, and triple anti-hypertensive groups. The mortality rate in groups taking ACEi/ARB was lower than other combination.

**Conclusions**: Hypertension can increase the severity of COVID-19. The use of ACEI/ARBs in ACE-2 receptor regulation which is thought to aggravate the condition of COVID-19 patients has not yet been proven. This is consistent with findings in other anti-hypertensive groups.

## Introduction

Coronavirus disease (COVID-19) had become a pandemic since almost one year ago. ( Huang *et al.*, 2020) Over 90 million cases and 1.5 million death-cases had happened during 2020 and several comorbidities associated with an increase in COVID-19 cases. Cardiovascular disease had become one of the most frequent comorbidities among COVID-19 patients which had a poor prognosis and high mortality rate. ( WHO, 2021)

The mechanism of severe acute respiratory syndrome coronavirus type 2 (SARS-CoV-2) infection is associated with the angiotensin-converting enzyme 2 receptor (ACE-2R) in the host cell and related with endothelial dysregulation. ( Saha *et al.*, 2021) Angiotensin-converting enzyme 2 (ACE2) plays a crucial role in renin-angiotensin-aldosterone system (RAAS) that regulate the blood pressure and main cause of hypertension. Hence, hypertension, and all complication-caused by hypertension, is associated with increased incidence and severity or risk of death in COVID-19. ( Ferrario *et al.*, 2005; Li *et al.*, 2020b)

One of the most common drugs for hypertension are angiotensin-converting enzyme inhibitors (ACEIs) and angiotensin receptor blockers (ARB). Some evidence had suggested the use of angiotensin-converting enzyme inhibitors (ACEIs) and angiotensin receptor blockers (ACEIs/ARB) in increasing the expression of ACE2 and hypothetically worsening the outcome of COVID-19 patients. ( Li *et al.*, 2020b; Ren *et al.*, 2021; Sanders *et al.*, 2020) Previous studies on several antihypertensive drugs had explained about the relation between increasing inflammatory markers, such as C-reactive protein, with the occurrence of cardiovascular disease. In COVID-19, there was an evidence of hyper-inflammation process that caused acute respiratory distress syndrome (ARDS) via the cytokine storm mechanism. ( Bas *et al.*, 2005; Moore & June, 2020) Therefore, in several studies, the use of anti-hypertensive drugs had become a debate in the occurrence of worsening disease progression in COVID-19 patients. ( Li *et al.*, 2020b; Li *et al.*, 2020d; Palvesky *et al.*, 2020; Parveen *et al.*, 2020; Ren *et al.*, 2021; Sunden-Cullberg, 2020; Zuin *et al.*, 2020) We hypothesize that the use of ACEI/ARBs might be correlated to ICU admission rate and length of stay, and may influence the inflammatory markers in COVID-19 patients.

Although several studies had associated few anti-hypertensive drugs to the severity and risk of death due to COVID-19 ( Li *et al.*, 2020c; Zuin *et al.*, 2020), we aim to test for an association between the use of anti-hypertensive drugs and the disease progression from moderate, severe, and critically ill COVID-19 patients in Universitas Airlangga Hospital (UAH). We used the UAH COVID-19 database that we had built since March 15, 2020.

## Methods

### Study design and population

We conducted a retrospective cohort study with consecutive sampling and eligibility criteria COVID-19 adult patients from moderate to critically ill patients admitted to Universitas Airlangga Hospital (UAH) Surabaya during March 15, 2020 to August 31, 2020 through hospital medical records. Universitas Airlangga Hospital is a teaching hospital and one of the referral hospitals for COVID-19 in Surabaya, East Java. Patients hospitalized in Universitas Airlangga Hospital are mostly grouped into moderate and critically-ill COVID-19. Therefore, our inclusive sample consisted of patients with a medical history of hypertension and previous medication with antihypertension, with moderate to critically ill COVID-19. Diagnosis of COVID-19 was made based on WHO guidelines and the Indonesian Ministry of Health guidelines, which were proven by oropharyngeal and nasopharyngeal swabs for SARS-CoV-2 PCR. Grouping of anti-hypertensive drugs based on three main groups ACEIs or ARBs (ACEI/ARB), calcium channel blockers (CCB) and beta blockers (BB) and we divided it into subgroups: single anti-hypertensive drugs, double anti-hypertensive drugs, and triple anti-hypertensive drugs. The group with multiple anti-hypertensive drugs consisted of patients with multiple diseases and uncontrolled hypertension, as narrated in Table 2.

**Figure 1.**
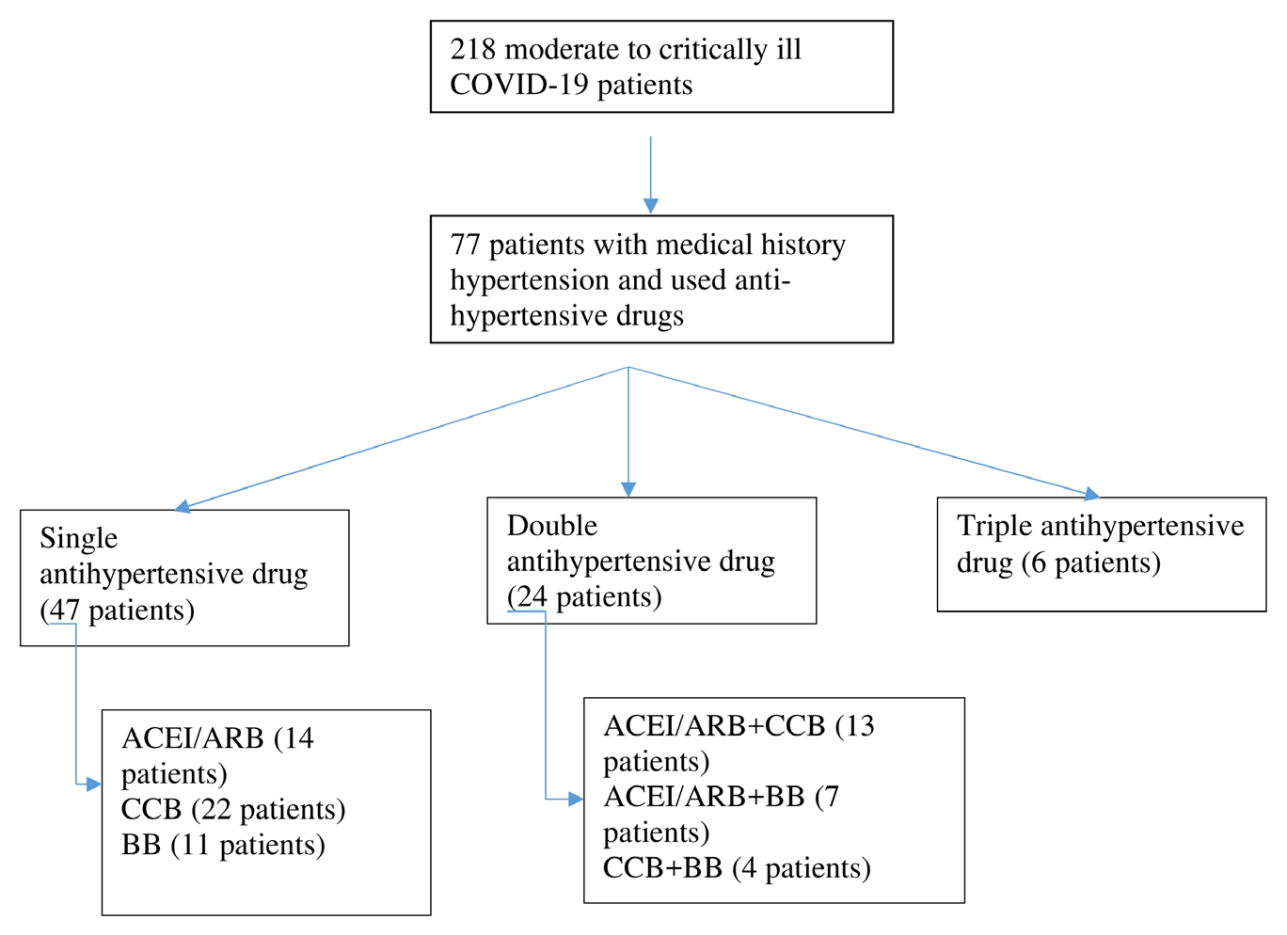
Selection of patients. ACEI/ARB: angiotensin-converting enzyme inhibitors and angiotensin receptor blockers; CCB: calcium channel blocker; BB: beta blocker.

The outcome of this study were mortality rate, ICU admission and days of death. The potential confounding was multiple comotbidities of population. To minimize bias we eliminated patient without a history of antihypertensive and had serial clinical and laboratory examinations until patient discharged or died. The study size was decribed at Figure 1. Serial chest x-ray and laboratory inflammatory marker evaluation was performed, and we grouped it into three periods of time evaluation. The first time was when the patient came to the hospital; means of second time evaluation was at day six, and the third time was when the patient was either discharged or died (mean time 13 days). 

### Data collection

Data was collected from medical records from March 15, 2020 to August 31, 2020. We had 218 patients with moderate to critically ill COVID-19 and medical records with incomplete clinical, laboratory, and radiology data were excluded. Clinical characteristics were divided according to the medical history of hypertension and anti-hypertensive drugs. We grouped anti-hypertensive drugs as single, double, and triple treatment. For single use anti-hypertensive drugs, we divided them into ACEI/ARB, CCB, and BB.

According to the 5th edition of COVID-19 National Guideline, moderate cases are defined as 1) clinical sign of pneumonia (fever, cough, dyspnea, tachypnea) and 2) Oxygen saturation ≥93% free air. Severe case definitions are if there were clinically sign of pneumonia, and one of this are the following: 1) respiration rate >30 times per minutes, or 2) severe respiratory distress, or 3) oxygen saturation < 93% free air. Moreover, the definition of critical conditions is when patients show symptoms of acute respiratory distress syndrome (ARDS) and septic shock ( Burhan *et al.*, 2020)

The severity score of COVID-19 pneumonia was assessed by simplifying the radiographic assessment of lung edema (RALE) score proposed by Warren *et al*. The RALE score assesses lung involvement into score zero as no involvement of the lung, score one for less than 25% involvement, score two for 25%–50% involvement of the lung, score three for 50%–75% involvement of the lung, and score four for more than 75% involvement of the lung. The results were divided into four categories: normal, mild (score one-two), moderate (score three-five), and severe (score six-eight). ( Warren *et al.*, 2018)

Inflammatory marker evaluation was based on the following criteria : 1) white blood cell count (WBC) >6.16x10 ^3 ^cells/μL; ( Feng *et al.*, 2020) 2) neutrophil-lymphocyte ratio (NLR) >6.5; ( Li *et al.*, 2020c) 3) Absolute lymphocyte count (ALC) < 1.0x10 ^3^ cells/ μL; ( Wagner *et al.*, 2020) 4) C-reactive protein (CRP) >41.8 mg/L; ( Liu *et al.*, 2020) and 5) procalcitonin (PCT) > 0,07 ng/mL. ( Liu *et al.*, 2020) 

This research was previously approved by the subjects with informed consent and was reviewed by ethical committee of Universitas Airlangga Hospital, Surabaya, Indonesia, number 179/KEP/2020. The study complies with the Declaration of Helsinki (Ethical Principles for Medical Research Involving Human Subjects) version 2013.

### Statistical analysis

Data were analyzed with SPSS version 24.0 (Chicago, IL, USA). Descriptive statistics included categorical variables reported as number (%) and continuous variables as mean (standard deviation). To control confounding, we did selection data by antihypertensive groups and severity. For missing data, we used list wise deletion or univariable and multivariable analysis. Chi-square test and Mann-Whitney test were applied according to the type of variable or subgroup interactions. Categorical variables were shown as number (%) and continuous variables as mean (standard deviation) or median (range) depending on whether the data are normally distributed. Statistical significance was assessed by means of chi-squared for dichotomous variables, or by means of the two independent sample t-test or the Mann-Whitney U test for continuous variable depending on whether the data are normally distributed. Analysis for the mean difference between the first, second and third evaluations of the lab results, radiology score severity, and length of stay using the ANOVA test. We calculated the risk ratio (RR) among subgroup analysis anti-hypertensive drugs to evaluate the outcome. Additionally, for the evaluation of disease progression among anti-hypertensive drugs group, we conducted a Cox-regression test. Kaplan-Meier curves were constructed for time survival in 30 days.

The strengthening the reporting of observational studies in epidemiology (STROBE) statement checklist was used for our report in this study ( Cuschieri, 2019).

## Results

### Patient characteristics

From 218 moderate to critically ill COVID-19 patients, 77 patients were inclusive of our criteria (see Figure 1). Forty-two patients were male (54.5%) and 35 were female (45.5%) with means aged 58.26 ± 12.59 years old. Although 55.84% of patients showed moderate symptoms, and based on RALE score, 46.75% were grouped to severe, we eventually still classified them referring to their clinical signs. The majority of comorbidities of the patients were hypertension (50.65%), diabetes mellitus (49.35%), geriatric (age > 60 years old) (40.26%), heart disease (10.39%), chronic kidney disease (7.79%), and obesity (2.6%).

Chief complaints in the admission such as dyspnea, fever, nauseous, vomit, diarrhea, malaise, runny nose and headache were mentioned. The findings of laboratory data showed that almost all inflammatory markers increased as could be seen in WBC counts 10,360/uL, neutrophil-lymphocyte ratio (NLR) 7.05, C-reactive protein (CRP) 72.5 mg/L, procalcitonin 4.48 ng/mL, creatinine serum 1.97mg/dL, and D-dimer 2.13 mcg/mL. We had been following all of the patients since the admission day up till the day of discharge, and data showed that the average length of hospital stay was 13.73 ± 6.69 days. During the follow up, 18 (23.4%) patients experienced deterioration in their condition and then they were admitted to the ICU, 15 patients (19.5%) had an acute respiratory distress syndrome (ARDS), and 12 (15,6%) were getting ventilated. For the characteristics of the patients see Table 1.

**Table 1.** Characteristics of patients. ACEI/ARB: angiotensin-converting enzyme inhibitors and angiotensin receptor blockers; CCB: calcium channel blocker; BB: beta blocker; ICU: intensive care unit; HT: hypertension.

**Gender**		
Male (n %)	42	54.5
Female (n %)	35	45.5
Age (mean/SD)	58.26	12.59
**Comorbidity**		
Geriatric (age>60 years old) (n %)	31	40.26
DM (n %)	38	49.35
HT (n %)	39	50.65
Heart Disease (n %)	8	10.39
Chronic Kidney Disease (n %)	6	7.79
Obesity (n %)	2	2.60
**Anti-Hypertensive Drugs**		
***Single***		
ACEI/ARB (n %)	14	18.18
CCB (n %)	22	28.57
BB (n %)	11	14.29
***Double***		
ACEI/ARB+CCB (n %)	13	16.88
ACEI/ARB+BB (n %)	7	9.09
CCB+BB (n %)	4	5.19
***Triple***		
ACEI/ARB+CCB+BB (n %)	6	7.79
**Severity of COVID-19**		
Moderate (n %)	43	55.84
Severe (n %)	9	11.69
Critically Ill (n %)	25	32.47
**Severity of chest x-ray**		
Normal (n %)	12	15.58
Mild (n %)	14	18.18
Moderate (n %)	14	18.18
Severe (n %)	36	46.75
**Symptom and duration**		
Dyspnea (% days)	42.9	3.1
Fever (% days)	49.4	2.79
Cough (% days)	59.7	5.79
Nausea and vomiting (% days)	14.3	4.1
Diarrhea (% days)	11.7	3.57
Malaise(% days)	18.2	3.64
Flu (%. days)	7.8	4.16
Headache (% days)	3.9	4
**Vital signs**		
Systolic pressure (mean SD)	140.78	26.78
Diastolic pressure (mean SD)	82.9	13.61
Pulse (mean SD)	101.88	15.46
Respiration rate (mean SD)	25.51	5.67
Temperature (mean SD)	36.96	0.92
Oxygen saturation (mean SD)	92.42	5.79
**Laboratory**		
Leucocyte (10 ^3^/uL; median SD)	8.89	6.16
Lymphocyte absolute (10 ^3^/uL median SD)	1.09	1.01
Neutrophil-lymphocyte ratio (NLR) (median SD)	5.56	4.63
Thrombocyte (10 ^3^/uL; median SD)	259	105.6
C-Reactive protein (mg/L; mean SD)	72.5	53.44
Procalcitonin (ng/ml; mean. SD)	4.48	10.8
Basal urea nitrogen (mg/dl; median SD)	15.5	29.92
Creatinine (mg/dl; median SD)	1.09	3.06
Aspartate aminotransferase (u/L; median SD)	22	310.22
Alanine aminotransferase (u/L; median SD)	15	115.07
D-Dimer (mcg/ml; mean. SD)	2.13	4.57
Glucose (mg/dl; mean SD)	198.38	130.72
PaO2:FiO2 ratio (median SD)	117.15	137.03
Length of Stay (mean SD)	13.73	6.69
ICU admission (n %)	18	23.4
ARDS (n %)	15	19.5
Ventilator (n %)	12	15.6
**Outcome**		
Discharge (n %)	58	75.3
Death (n %)	12	15.6

DM, diabetes mellitus; HT, hypertension; ACE, angiotensin-converting enzyme inhibitors; ARB, angiotensin receptor blockers; BB, beta blocker; CCB, calcium channel blocker; ICU, intensive Care Unit; SD, standard deviation.

### Laboratory and radiology characteristics

Based on the characteristics, each group of anti-hypertensive drugs had comorbidities. In the single anti-hypertensive group, patients grouped in the CCB and BB had several significant comorbidities, e.g. elderly>60 years, diabetes mellitus, and hypertension. Among those three groups of single anti-hypertensive therapy, patients grouped in the ACEI/ARB and BB had controlled blood pressure. Differences in systolic blood pressure of patients were discovered among these three groups, which were 136.43 mmHg in ACEI/ARB group, 142.91 mmHg in CCB groups, and 117.91 mmHg in BB group (p 0.05), whereas other clinical parameters such as diastolic blood pressure, pulse, respiration rate, temperature and oxygen saturation showed no significant differences. Radiological evaluation showed that ACEI/ARB, CCB, and BB group were in the moderate category, although ACEI/ARB seemed to have the lowest radiological severity score.

An increase of the inflammatory markers such as WBC count, NLR, ALC, CRP, PCT creatinine serum, and D-dimer were recorded after periodical follow-ups of all groups. ACEI/ARB group showed the elevation of white blood cell counts along with the CRP and PCT. It might be hypothesized that the initial character of patients in ACEI/ARB group had previously high numbers of inflammatory markers with the possibility of secondary infection. ACEI/ARB also did not deteriorate the renal function of moderately to critically ill patients who were prone to experience systemic vasodilation. This was indicated by the return of serum creatinine after episodes of acute kidney injury in the third treatment evaluation.

The WBC and CRP counts in CCB group were only slightly detected if compared to other two groups. This might be related to a low level of procalcitonin at two previous evaluations. However, the group treated with CCB was more susceptible to developing acute kidney injuries as evidenced by the increase in serum creatinine levels. Patients with COVID-19 being treated with BBs also showed the highest numbers of some inflammatory markers such as NLR, CRP, and SCr in the second evaluation. The major comorbidities of the BB-treated group were diabetes mellitus (DM) type 2 (54.55%), hypertension (HT) (36.36%), and geriatic (age > 60 years old) (36.36%), and minor comorbidities were heart disease and obesity. (9.09%)

Patients taking double anti-hypertensive agents were also categorized into ACEI/ARB and CCB group, ACEI/ARB and BB group, and CCB and BB group. In groups involving ACEI/ARB, laboratory markers showed lower level of NLR (3.13 and 6.15), than in groups taking BB and CCB (12.04). Otherwise, D-dimer levels surged in the group of patients taking BB and CCB.

We then expanded the group into larger ones of patients taking single, double and anti-hypertension groups. Elderly patients, diabetes mellitus, and hypertension were comorbidities found in all these three groups. In accordance with the characteristics of blood pressure, patients who took more anti-hypertension drugs had poorer blood pressure control, patients in the single anti-hypertension group presented 135.13 mmHg of systolic blood pressure, 147.13 mmHg in the double anti-hypertension group and 159.67 mmHg in the triple anti-hypertension group and were statistically different (p 0.038). Significant differences between groups were also seen in the CRP results at baseline admission (p 0.005) and the first evaluation of oxygen fraction (p 0.032).

### Outcome associated to anti-hypertension

**Figure 2.**
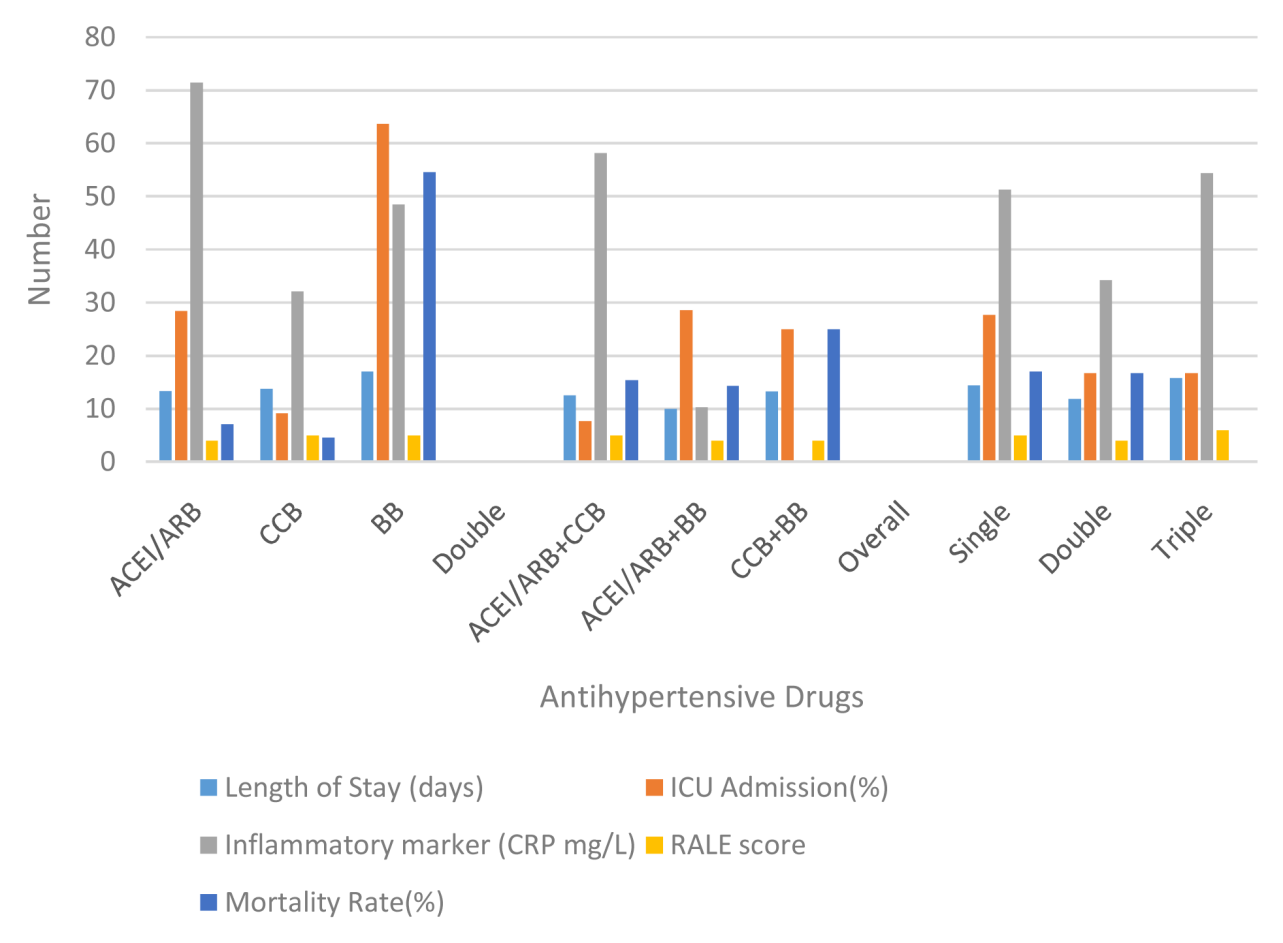
Comparison between group anti-hypertensive drugs. ACEI/ARB: angiotensin-converting enzyme inhibitors and angiotensin receptor blockers; CCB: calcium channel blocker; BB: beta blocker; ICU: intensive care unit; RALE: radiographic assessment of lung edema.

Based on the inflammatory markers and evaluation on the case severity using x-ray follow ups, it is crucial to discuss the effect of hypertension to the clinical outcome of patients with COVID-19. Based on the single anti-hypertension analysis, patients who received only BBs had a longer length of stay than ACEI/ARB or CCB alone (17, 13.36, and 13.73 respectively). After the evaluation, patients who received anti-hypertension BBs also had the highest rates of ICU admission (63.64). This rate is quite high in number when compared to ACEI/ARB or CCB (p 0.04). Nevertheless, although ACEI / ARB had a fairly high rate for ICU admission (28.57), the discharge rate was also quite high (71.43%) with a mortality rate of 7.14%. The highest mortality was found in the BB group (54.55%) with a discharge rate of 45.45% (p 0.032) (see Table 2 and Figure 2).

**Table 2.** Evaluation anti-hypertensive drugs in COVID-19 patients. ACEI/ARB: angiotensin-converting enzyme inhibitors and angiotensin receptor blockers; CCB: calcium channel blocker; BB: beta blocker.

Single												Double												Single			Double			Triple		
ACEI/ARB			CCB			BB			pValue			ACEI/ARB+CCB			ACEI/ARB+BB			CCB+BB			pValue											
14			22			11			47			13			7			4			24			47			24			6		
42,86			45,45			36,36						30,77			42,86			25,00						42,55			33,33			50,00		
28,57			45,45			54,55						61,54			85,71			0,00						42,55			58,33			66,67		
50,00			40,91			36,36						53,85			85,71			50,00						42,55			62,50			66,67		
21,43			4,55			9,09						0,00			14,29			0,00						10,64			4,17			33,33		
0,00			4,55			0,00						7,69			0,00			50,00						2,13			12,50			33,33		
0,00			0,00			9,09						0,00			14,29			0,00						0,00			0,00			0,00		
136,43			142,91			117,91			0,05			152,92			142,29			136,75			0,32			135,13			147,13			159,67		
82,29			83,41			74,73			0,22			86,23			88,00			83,00			0,81			81,04			86,21			84,17		
98,07			101,18			101,73			0,78			95,46			113,71			101,25			0,043 *			100,38			101,75			114,17		
25,00			24,76			29,55			0,09			23,46			25,29			24,00			0,57			25,98			24,08			27,67		
36,93			37,38			36,64			0,09			36,85			36,57			36,50			0,63			37,07			36,71			37,17		
92,21			92,27			92,45			0,99			93,00			90,00			94,75			0,53			92,30			92,42			93,33		
13,36			13,73			17			0,332			12,54			10			13,25			0,486			14,38			11,92			15,83		
28,57			9,09			63,64			0,04 *			7,69			28,57			25,00			0,43			27,66			16,67			16,67		
71,43			86,36			45,45			0,032 *			76,92			85,71			50,00			0,82			72,34			75,00			100,00		
7,14			4,55			54,55						15,38			14,29			25,00						17,02			16,67			0		
ACEI/ARB			CCB			BB			pValue			ACEI/ARB+CCB			ACEI/ARB+BB			CCB+BB			pValue			Single			Double			Triple		
1	2	3	1	2	3	1	2	3	1	2	3	1	2	3	1	2	3	1	2	3	1	2	3	1	2	3	1	2	3	1	2	3
5	3	4	5	5	5	5	5	5	0,87	0,41	0,39	4	4	5	4	5	4	3	4	4	0,83	0,85	0,95	5	4	5	4	4	4	4	4	6
15,48	13,80	11,02	8,46	8,36	9,93	9,95	13,67	20,47	0,012 *	0,042 *	0,015 *	7,45	9,15	9,39	10,54	13,72	15,03	9,70	8,97	11,32	0,17	0,28	0,10	10,90	11,12	13,14	8,73	10,42	11,06	12,65	13,99	10,98
1409,98	887,48	907,88	1194,26	957,58	927,80	1201,26	1108,56	1027,68	0,64	0,74	0,95	1208,42	1012,31	1534,75	2374,58	1316,24	794,55	829,08	1353,15	888,78	0,08	0,57	0,34	1260,15	972,04	945,24	1485,33	1157,77	1211,20	1963,14	1183,51	920,11
10,31	8,06	3,41	5,91	5,94	3,94	7,20	10,18	12,64	0,014 *	0,35	0,06	4,83	5,77	3,14	4,96	7,67	6,16	12,02	9,28	12,04	0,024 *	0,77	0,14	7,52	7,56	5,82	6,07	6,91	5,50	7,39	9,01	5,05
249,57	249,10	301,63	255,55	348,44	375,62	260,09	368,67	220,75	0,95	0,10	0,04	296,23	374,27	381,50	288,29	340,17	398,25	384,25	297,00	294,33	0,55	0,65	0,65	254,83	326,51	312,48	308,58	349,81	370,06	300,83	382,40	470,25
20,53	36,46	71,40	13,91	3,00	32,07	0,50	48,45	9,99	0,49	0,79	0,87	3,01	10,30	58,20	9,89		10,30	6,07			0,68			13,58	34,56	51,25	5,63	10,30	34,25	60,10	86,70	54,42
3,29	2,46	8,32	0,27	0,76	0,43	2,67	2,25	8,89	0,45	0,61	0,60	0,02	1,56	40,14	0,25	0,05		0,03	0,05	0,24	0,16	0,82	0,72	1,71	1,97	5,66	0,09	1,18	32,16	5,09	0,38	
18,13	27,90	42,97	15,67	16,69	20,14	26,76	25,25	56,49	0,14	0,38	0,33	30,92	13,69	17,99	21,10	29,13	27,87	79,50	51,35	47,05	0,12	0,03	0,23	18,83	22,34	39,58	36,15	26,00	24,74	21,43	52,17	30,00
0,99	16,30	1,25	1,81	1,47	2,22	1,41	1,41	1,73	0,52	0,35	0,68	2,37	1,34	1,61	1,61	0,90	1,30	6,61	4,63	4,24	0,14	0,10	0,23	1,47	6,05	1,78	2,85	2,08	1,94	2,36	2,43	1,07
48,69	47,17	49,80	22,20	36,60	40,00	326,67	86,33	54,00	0,15	0,17	0,78	26,08	51,29	21,50	50,86	76,00	38,00	33,50	567,00	132,00	0,59	0,08	0,21	95,64	57,88	47,00	34,91	149,55	47,89	31,00	27,00	33,00
33,15	95,50	111,40	21,00	37,40	55,57	148,67	216,17	118,80	0,07	0,37	0,65	27,33	95,14	32,17	46,86	116,00	114,00	21,00	247,00	30,00	0,68	0,33	0,017 *	52,12	121,00	90,59	32,17	126,55	40,78	19,50	23,67	33,00
1,52	2,15	2,20	5,93	3,93	1,86	5,88	7,31	1,87	0,34	0,37	0,94	1,49	2,52	1,31	1,52	3,10	0,00	0,00		11,16	0,31	0,52	0,16	5,04	4,45	1,93	1,44	2,61	4,59	7,93	5,25	4,77
131,72	427,33	490,00	210,43	367,25	285,50	141,03	324,36	401,75	0,33	0,21	0,67	165,66	385,00	272,00	192,60	568,75	326,50	99,08	375,00	669,00	0,72	0,37	0,30	161,63	358,68	398,25	161,02	488,57	452,60	91,08	573,00	396,50

ACE, angiotensin-converting enzyme inhibitors; ARB, angiotensin receptor blockers; BB, beta blocker; CCB, calcium channel blocker; RALE, radiographic assessment of lung edema WBC, white blood cells; ALC, absolute lymphocyte count; NLR, neutrophil to lymphocyte ratio; Plt, platelet; CRP, C-reactive protein; PCT, procalcitonine; BUN, blood urea nitrogen; SCr, serum creatinine; SGOT, Serum Glutamic Oxaloacetic Transaminase; SGPT, Serum Glutamic Pyruvic Transaminase; P/F ratio, PO2/FiO2 ratio; * : p-Value < 0.05

**Figure 3.**
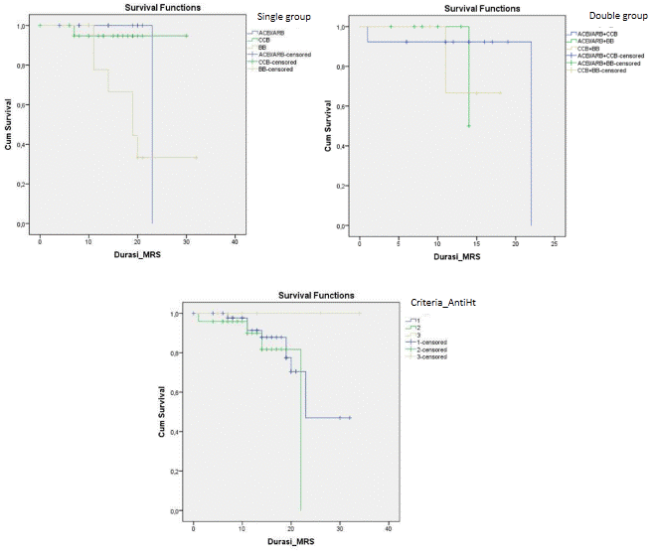
Time survival in 30 days among antihypertensive drugs. ACEI/ARB: angiotensin-converting enzyme inhibitors and angiotensin receptor blockers; CCB: calcium channel blocker; BB: beta blocker.

Disease progression can be observed through number patients died and days of death (time from first admission to death) from comorbidity, anti-hypertensive drugs, severity of chest x-ray, and inflammatory markers. It showed that heart disease (25%) and being geriatric (19.35%) contributed to a higher mortality rate than the others’ comorbidities. Nevertheless, all comorbidities were not significantly different for the days of death. No mortality had occurred in patients with CKD. Compared to the anti-hypertensive drugs used, there was significance in single use BBs for mortality events and days of death (RR: 9.82; 95% CI: 2.3–41.89; HR: 6.64; 95% CI: 1.3–33.04) (see Table 3 and Figure 3). Comparing single, double and triple anti-hypertensive drugs groups, there were no significant differences in length of stay, ICU admission, mortality rate, and days of death. Even though patients taking ACEI/ARB in both the single and combination groups had quite a high number of inflammatory marker variables, the mortality rate was still lower when compared to other anti-HT drug combinations. This suggests that ACEI/ARB has a protective effect (see Table 2 and Table 3). Additionally, more severe chest x-ray findings and higher inflammatory markers correlated to a higher mortality rate but had no significant effect on days of death.

**Table 3.** Disease progression among anti-hypertensive drugs. ACEI/ARB: angiotensin-converting enzyme inhibitors and angiotensin receptor blockers; CCB: calcium channel blocker; BB: beta blocker; CI: confidence interval.

Comorbid	Mortality rate(%)	Days of death (mean)	RR	95%CI
Geriatri (age>60 years old)	19,35	23,52		
DM	15,79	27,91		
HT	17,95	25,98		
Heart Disease	25,00	22		
Chronic Kidney Disease	0,00	0		
AntiHypertensive Drugs				
Single				
ACEI/ARB	7,14	23	0,33	(0,05-2,49)
CCB	4,55	28,79	0,16	(0,02-1,22)
BB	54,55	21,11	9,82	(2,33-41,89) *
Double				
ACEI/ARB+CCB	15,38	20,39	0,85	(0,14-5,06)
ACEI/ARB+BB	14,29	14	0,81	(0,10-6,51)
CCB+BB	25	15,67	1,27	(0,17-9,72)
Overall				
Single	17,02	25,19	1,28	(0,42-3,49)
Double	16,67	19,81	1,10	(0,37-3,31)
Triple	0	0		
Severity of Chest X-Ray				
Normal	0	0		
Mild	7,14	19		
Moderate	14,29	18		
Severe and Critcal ill	24,32	25,03		
Inflammatory marker				
Leucocyte				
>6.16x10 ^3^/uL	12,99	26,22		
<6,16x10 ^3^/uL	2,60	21,27		
Neutrophil-lymphocyte ratio (NLR)				
High (>6.5)	7,79	25,88		
Low (<6,5)	7,79	24		
Absolute Lymphocyte Count (ALC)				
Low < 1.0 × 10 ^3^ cells/µL	7,79	23,54		
High > 1.0x10 ^3^ cells/µL	7,79	27,31		
CRP				
High (>41,8 mg/L)	6,49	23,26		
Low (<41,8 mg/L)	9,09	27,64		
Procalcitonin				
High (>0,07 ng/mL)	9,09	23,96		
Low (<0,07 ng/mL)	6,49	28,07		

DM, diabetes mellitus; HT, hypertension; ACE, angiotensin-converting enzyme inhibitors; ARB, angiotensin receptor blockers; BB, beta blocker; CCB, calcium channel blocker; ICU, intensive Care Unit; SD, standard deviation; NLR, Neutrophil-lymphocyte ratio; ALC, Absolute Lymphocyte Count; CRP, C- Reactive Protein; * : marked significantly according 95% confidence interval.

## Discussion

According to the results of our study, COVID-19 confirmed cases may appear with multiple comorbidities, inducing significant inflammatory markers that affect the clinical outcomes of the patients. We analyzed the results by grouping the antihypertension agents into three groups: ACEI/ARB, CCB, and BB. Hypertension is believed to be one of the most common comorbidities. Patients involved in this research had a medical history of hypertension and was previously given the anti-hypertension agent before or during the hospital care. Most of the patients with a previous history of hypertension along with other comorbidities, such as heart failure, diabetes mellitus and received BBs. Although BB seems to be frequently used by patients with heart failure, it is also vastly used to lower the blood pressure in diabetes mellitus patients.

COVID-19 has been known to stimulate an inflammation cascade. A preceding result states that approximately one-fourth of patients with septic condition have lymphopenia. Addtionally, lower absolute lymphocyte count (ALC) (< 1.0x10 ^3^ cells/μL) could be a prognostic factor for COVID-19. ( Giamarellos-Bourboulis *et al.*, 2020; Wagner *et al.*, 2020)

It has been shown that proinflammatory cytokines and chemokines including tumor necrosis factor (TNF) α, interleukin 1β (IL-1β), IL-6, granulocyte-colony stimulating factor, interferon gamma-induced protein-10, monocyte chemoattractant protein-1, and macrophage inflammatory proteins 1-α were significantly elevated in COVID-19 patients. It is characterized by sustained and substantial reduction of the peripheral lymphocyte counts, mainly CD4-T and CD8-T cells in COVID-19 patients, and is associated with a high risk of developing secondary bacterial infection. ( Li *et al.*, 2020a)

Our study showed that high levels of inflammatory markers, such as WBC, PCT, and NLR, were correlated with higher mortality rates but showed no diffrerences for days of death. CRP levels were higher in the ACEI/ARB group, both single and double, but the mortality rate wasn’t higher than BB groups.

Hypertension, as stated by the American Heart Association, is defined as the rise of systolic blood pressure to the point of 140 mmHg and above, and/or diastolic blood pressure to 90mmHg and above. ( PERHI, 2019) Hypertension is the leading cause of mortality globally. Approximately one-third of adults were estimated to have hypertension worldwide in 2010. Since the emerge of SARS-CoV-2 infection, hypertension has been frequently observed as a major comorbidity in patients with COVID-19. ( Casucci *et al.*, 2020; Yang *et al.*, 2020) SARS-CoV2 virus penetrates into the cell through the ACE2 receptor. ACE2 was recognized as an enzyme having a role in the renin-angiotensin system (RAS) regulation. The transport of ACE2 to SARS-Cov-2 virus escalates the activity of the protein containing disintegrin domain and metalloproteinase domain-containing protein 17 (ADAM17). ADAM17 induces the release of ACE-2 ectodomain and produces soluble ACE-2 in the blood circulation. The low expression of ACE2 in the cell surface might lower the possibility for the infection to occur. The strong inhibition of ACE2 by SARS-CoV2 leading to increased vascular permeability and pulmonary edema ( Yang *et al.*, 2020). Early investigations in SARS-CoV suggest that ACE2 may have both a pathogenic role in facilitating virus infection and a protective effect in limiting lung injury during SARS-CoV-2 infection. As we know, ACE2 is recognized as a critical enzyme that regulates blood pressure, fluid and electrolyte balance, and vascular resistance by renin-angiontensi-aldosterone system. Drugs that can upregulate the expression of ACE2, such as ARBs and ACE inhibitors, have been commonly used in patients with hypertension and other cardiovascular diseases to regulate blood pressure and reduce the mortality and morbidity events ( Gul *et al.*, 2021; Shibata *et al.*, 2020; Yang *et al.*, 2020).

The mechanism of hypertension in worsening the clinical outcome has not been yet explained. Several studies declared that ACEI-induced angiodema was associated with increasing CRP and BB was associated with lower CRP level. Nevertheless, Yang *et al.* declared that patients with hypertension came with highly detected inflammatory markers such as CRP (p=0.024), Procalcitonin (p=0.017) and IL6 (p=0.017). Angiotensin II is a renin-angiotensin effector peptide, responsible in pro-inflammatory cytokines induction, e.g IL-6, IL-1 β, TNF-α, IFN-ϒ, IL-17 and IL-23. Therefore, ACE inhibitors and ARBs that are capable of reducing the production of inflammatory cytokines are potential candidate drugs for treatment of patients with COVID-19 and preexisting hypertension. ( Bas *et al.*, 2005; Palmas *et al.*, 2007; Yang *et al.*, 2020 )

Based on the known SARS-Cov-2 pathogenesis, there seems to be a strong correlation among hypertension, anti-hypertensive agents, and SARS-Cov-2 infection. The SARS-CoV-2 receptor binds to the angiotensin-converting enzyme 2 (ACE-2) found on the respiratory cells with the help of S protein, which helps in entry and the replication process. After the multiplication process, when the virus reaches the lungs, it causes inflammation in the alveoli or lung sacs leading to pneumonia. Several patients would suffer from severe sepsis, shock, and even ARDS. ( Kumar *et al.*, 2020)

This research shows that the final clinical outcome from each of the group; ACEI/ARB, CCB, and BB might vary despite the similar initial condition. It also has been observed that the patients’ discharge and mortality numbers among these three groups show significant different results. Data shows that the discharge numbers of ACEI/ARB group are high (71.43%) while the ICU admission rate reached a third of the BB group. Comorbidities also play a role in severity and higher mortality rate among groups but isn’t different in days of death.

This result was similiar with the Ren *et al.* study, which showed that the use of antihypertensive drugs weren’t correlated with the severity and risk of COVID-19. ( Ren *et al.*, 2021) Compared to previous local and regional studies, we didn’t find any study that compared antihypertensive drugs with disease progression in COVID-19. 

The use of ACEI/ARB is still a matter of discussion due to the ACE-2 numerous receptor theories. ARBs/ACE inhibitors treatment has been reported to increase the expression of ACE2, which is also the cellular receptor for SARS-CoV-2 infection. It was suggested a protective role of ACE2 upregulation and ARBs/ACE inhibitors treatment in COVID-19 and raised concerns that ARBs/ACE inhibitors treatment could promote SARS- CoV-2 infection by increasing the expression of ACE2. ( Yang *et al.*, 2020)

Despite many hypotheses, evidence from a series of cohort studies published recently suggests that previous or current treatment with ACEIs or ARBs does not increase the risk and the complication of COVID-19 infection. ACEI and ARB act by inhibiting the renin-angiotensin-aldosterone system (RAAS). Angiotensinogen is converted to angiotensin I (AngI) by renin, then converted to angiotensin II (AngII) by angiotensin converting enzyme (ACE). Furthermore, angiotensin is converted to angiotensin (1–7) by angiotensin converting enzyme-2 (ACE-2). Angiotensin II activates angiotensin 2 type 1 receptor (AT1R) which stimulates vasoconstriction, pulmonary edema, oxidative effects, inflammation, and fibrosis. Meanwhile, angiotensin 1–7 have anti-inflammatory, anti-apoptotic, and anti-fibrosis effects. Both ACEI and ARB inhibit the ACE, hence the amount of ACE will be detected abundantly in the circulation. ACE in the systemic circulation is thought to have protective effects on the lungs, particularly in preventing the ARDS. At the same time, a decrease in ACE activity will increase Ang II levels, causing the activation of the AT1 receptor that produces ARDS. ( Gul *et al.*, 2021; Saha *et al.*, 2021; Yang *et al.*, 2020) 

On the other hand, a study by Trump *et al.* comparing the proportion of critical cases to all other severities of COVID-19 in the different patient groups with hypertension and cardiovascular diseases that receive anti-hypertensive agents, show that ACEI treatment proves to have a more profound decline in critical cases compared to other antihypertensive agent. Also, the results successfully showed the overall increased expression of both *ACE2* ( *P* = 0.0025) and *TMPRSS2* ( *P* = 0.0002) upon SARS-CoV-2 infection. However, anti-hypertensive treatment did not alter ACE2 expression, in neither patient positive for SARS-CoV-2 nor patients negative for SARS-CoV-2. Therefore, entry factor expression did not predispose ACEI or ARB-treated patients to SARS-CoV-2 infection. This finding is in accordance with observational studies, which did not reveal any effect of ACEI or ARB treatment on SARS-CoV-2 infection risk in individuals with hypertension or other cardiovascular diseases. ( Trump *et al.*, 2020)

In accordance with the guidelines of the European Society of Cardiology, there is no change in anti-hypertensive recommendations for COVID-19 patients. CCBs, both non-dihydropyridine and dihydropyridine can still be considered. Verapamil, a non-dihydropyridine CCB, is able to control heart rate in supraventricular tachycardia (SVT), prevent the migraine attack, and to manage the high blood pressure in patients with atrial fibrillation. This drug can be the drug of choice in hypertension management in COVID-19 patients. Preliminary data from animal studies suggest that verapamil has no effect on ACE2 expression, cardiac involvement, and SARS-Cov-2 related myocarditis. Amlodipine, a CCB dihydropyridine, shows some benefits in COVID-19 patients by inhibiting SARS COV-2 infection *in vitro* through the role of intracellular Ca2+, inhibiting viral replication at the post entry stage, showing inhibitory effects against SARS-Cov2 replication, and enhancing the anti-viral effect of chloroquine. ( ESC, 2020)

The use of BBs also showed promising benefits in patients with COVID-19. Beta-adrenergic blockers block the entry of SARS-COV-2 via the ACE2 receptor as well as CD147. BBs on the juxtaglomerular cells in the kidney reduce the activity of both arms of the RAAS pathway, thereby it may decrease the ACE2 level. As ACE2 is the receptor for SARS-CoV-2 cellular entry, beta-adrenergic blockers may decrease the SARS-CoV-2 cellular entry. Therefore, beta-adrenergic blocker treatment in COVID-19 will decrease the SARS-CoV-2 cellular entry by downregulation of both ACE2 and CD147. Beta-adrenergic blockers have been shown to decrease a variety of proinflammatory cytokines expression including IL-1β, IL-6, TNFα, IFNγ. The use of beta-adrenergic blockers in COVID-19 patients may reduce the expression of the proinflammatory cytokines and the inflammation associated with it. ( Vasanthakumar, 2020)

Our study showed that patients who were treated with single BB had higher ICU admission, mortality rate, and shorten days of death when compared with the others who were treated with only anti-hypertensive drugs. Nevertheless, when compared to double and triple antihypertensive drugs, as a single group, there was no significant difference.

There are some limitations of our study. First, we only used retrospective data from medical records. Second, the ratio of the population involved in the study using anti hypertension BB is quite small so it might not reflect the general population. Third, there may be an effect of multiple comorbidities in the study population which has the potential to be confounder which may effect the study results.

## Conclusion

Hypertension is one of the comorbidities that causes increased severity of COVID-19. The effect of ACEI or ARBs on ACE-2 receptor regulation, which is thought to aggravate the condition of COVID-19 patients has not yet been proven. This is consistent with findings in other anti-hypertensive groups.

## Data availability

### Underlying data

Figshare. Data of Anti Hypertensive in COVID-19. DOI: 10.6084/m9.figshare.14130584

This project contains the following underlying data:

- This .xls dataset contains patients’ history, physical examination, laboratory data, and chest x-ray imaging.

Data are available under the terms of the Creative Commons Attribution 4.0 International license (CC-BY 4.0).
