# Supplementary material for: Correlation between anti-hypertensive drugs and disease progression among moderate, severe, and critically ill COVID-19 patients in the second referral hospital in Surabaya: A retrospective cohort study
Source: F1000Res. 2023 Jul 21;10:393. Originally published 2021 May 17. [Version 3] doi: 10.12688/f1000research.51785.3 (PMC8593622; doi:10.12688/f1000research.51785.3)
Supplement: Peer review history

# Peer review: Reviewer response for version 2

**DOI:** 10.5256/f1000research.77693.r95399

**Published:** 2021-11-15

**Authors:** Bertram Pitt

**Affiliations:** 1 Department of Medicine, University of Michigan School of Medicine, Ann Arbor, MI, USA

The authors have responded to my suggestions, and I believe the paper is now acceptable for indexing.

Is the work clearly and accurately presented and does it cite the current literature?

Yes

If applicable, is the statistical analysis and its interpretation appropriate?

No

Are all the source data underlying the results available to ensure full reproducibility?

Partly

Is the study design appropriate and is the work technically sound?

Partly

Are the conclusions drawn adequately supported by the results?

Partly

Are sufficient details of methods and analysis provided to allow replication by others?

Yes

Reviewer Expertise:

Cardiology; heart failure; RAAS; CKD; hypertension; diabetes

I confirm that I have read this submission and believe that I have an appropriate level of expertise to confirm that it is of an acceptable scientific standard.

# Peer review: Reviewer response for version 2

**DOI:** 10.5256/f1000research.77693.r95400

**Published:** 2021-10-04

**Authors:** Dhite Bayu Nugroho, Mawaddah Ar Rochmah

**Affiliations:** 1 Facuty of Medicine, Public Health and Nursing, Universitas Gadjah Mada, Yogyakarta, Indonesia

The author has made changes to the manuscript in response to the reviewers' suggestions. This new version is preferable to the previous version and merits indexing.

Is the work clearly and accurately presented and does it cite the current literature?

Yes

If applicable, is the statistical analysis and its interpretation appropriate?

Partly

Are all the source data underlying the results available to ensure full reproducibility?

Yes

Is the study design appropriate and is the work technically sound?

Partly

Are the conclusions drawn adequately supported by the results?

Partly

Are sufficient details of methods and analysis provided to allow replication by others?

Yes

Reviewer Expertise:

Cardiovascular, adipose tissue angiogenesis, cardiac hypertrophy, Senescence, Endothelin, Adipokine

We confirm that we have read this submission and believe that we have an appropriate level of expertise to confirm that it is of an acceptable scientific standard.

# Peer review: Reviewer response for version 1

**DOI:** 10.5256/f1000research.54990.r85479

**Published:** 2021-07-02

**Authors:** Bertram Pitt

**Affiliations:** 1 Department of Medicine, University of Michigan School of Medicine, Ann Arbor, MI, USA

This is a well written review of the role of antihypertensive medication in patients with COVID-19. There have however been several prior papers pointing out the role of hypertension in COVID-19 and the relative risk of various antihypertensive medications. It would therefore be important to point out what new information this paper provides.

 It might also be of value to discuss the role of obesity, since obesity is a risk factor for COVID-19 and often co-exists with hypertension.

 In regards to the the effect of the individual classes of antihypertensive medications in patients with COVID-19, it should be emphasized that the number of patients is rather small and that therefore no definitive conclusions can be drawn. It might be of interest to present statistical power calculations for the sample size that would be needed to reach a significant conclusion in regard to the risk of the individual agents in patients with COVID-19.

 Without further explanation of what is new in this paper to justify publication, I do not believe it has sufficient priority for indexing.

Is the work clearly and accurately presented and does it cite the current literature?

Yes

If applicable, is the statistical analysis and its interpretation appropriate?

No

Are all the source data underlying the results available to ensure full reproducibility?

Partly

Is the study design appropriate and is the work technically sound?

Partly

Are the conclusions drawn adequately supported by the results?

Partly

Are sufficient details of methods and analysis provided to allow replication by others?

Yes

Reviewer Expertise:

Cardiology; heart failure; RAAS; CKD; hypertension; diabetes

I confirm that I have read this submission and believe that I have an appropriate level of expertise to confirm that it is of an acceptable scientific standard, however I have significant reservations, as outlined above.

# Peer review: Reviewer response for version 1

**DOI:** 10.5256/f1000research.54990.r85528

**Published:** 2021-06-04

**Authors:** Dhite Bayu Nugroho, Mawaddah Ar Rochmah

**Affiliations:** 1 Facuty of Medicine, Public Health and Nursing, Universitas Gadjah Mada, Yogyakarta, Indonesia

**General comments:**

 This study talks about the difference in antihypertension drugs (AHD) used in COVID-19 patients with hypertension. Although similar studies have been published, this study emphasizes that the use of ACEI may not differ from other AHD's in the outcome of COVID-19.

 Although this might give merit to the knowledge of COVID-19, several issues need to be addressed:

1. The criteria of COVID-19 severity in this study should be clearer and must refer to an acceptable recommendation/guideline. Please provide the reference.
2. The difference with other similar studies should be stated clearly.
3. Authors are advised to go through the manuscript again to correct the grammatical errors. A language edit is suggested.

**Specific comments:**

 Page 3

- What is the impact of UAH being a referral hospital? Did it affect the severity of the admitted patients? Please explain.
- Is previous medication with antihypertension drugs also accounted for the inclusion and classification of the patient?
- *"Coronavirus disease (COVID-19) had become a pandemic since almost one year ago. (Huang et al., 2020) Over 90 million cases and 1.5 million death-cases had happened during 2020 and several comorbidities associated with an increase in COVID-19 cases. Cardiovascular disease had become one of the most frequent comorbidities among COVID-19 patients which had a poor prognosis and high mortality rate. (WHO, 2021)"* - This paragraph should be revised for its grammatical errors. The authors are advised to use the past tense. Because the data related to this pandemic is rapidly changing, the authors should reference when this data was obtained.
- *"Several antihypertensive drugs had been studied about the relation of increasing inflammatory markers like C-reactive protein in cardiovascular disease" - *This sentence is confusing. Please revise.
- *"The critical condition is upright when symptoms are present that supports the diagnosis of acute respiratory distress syndrome (ARDS) and septic shock (Burhan et al., 2020)." -* What does it mean by "upright"?
- *"For severe COVID-19, moderate case definitions were defined as: 1) clinical sign of pneumonia (fever, cough, dyspnea, tachypnea) and 2) Oxygen saturation ≥93% free air. Severe case definitions are if there were clinically sign of pneumonia, and one of this are the following: 1) respiration rate >30 times per minutes, or 2) severe respiratory distress, or 3) oxygen saturation < 93% free air."* - Please insert citations that recommend this classification of COVID-19 severity.
- *"Although several studies had associated few antihypertensive drugs to the severity and risk of death due to COVID-19,..." - T*his sentence needs references. What is the difference between this study and other similar ones?

Page 4

- "Figure 1", please explain the abbreviation of "BB". Please explain the types of antihypertensive agents in the group receiving triple agents and The rationale for using more than 1 AHD?
- *"Although 55.84% severity of COVID-19 was moderate but based on RALE score 46.75% were included in severe and critically ill." - *Thus, which criteria were used for the classification of moderate vs severe COVID-19? Is it clinical or radiological criteria?
- What about other medications/ co-medications? Were all subjects received the same medications for COVID-19 other than the antihypertension drugs? Please state it here.
- In the descriptive analysis, data that are not normally distributed should be shown as the median.
- *"The strengthening of the reporting of observational studies in epidemiology (STROBE) statement checklist was used for our report in this study."* - Please add references.

Page 5

-  "Table 1" - Please give appropriate subtitle for each column. I think "sex" is not the best subtitle for this part.

Page 6-8

- "Table 2", a significant p-value, should be marked.
- "Table 2" - Please explain why there is a missing p-value in table 2 in comparing the use of Antihypertensive agents in the comorbidity group.
- Please explain why the discharge and death outcome parameters have the exact p-value. What variables are the researchers trying to compare on this outcome parameter?
- A lot of information in this manuscript refers to the table on page 8 of the pdf version. However, this table is not clearly explained: is this table part of table 2? With different types of information, this table should be dedicated as a separate table. What is meant by evaluations 1,2, and 3? The information displayed is too convoluted, although not much of this information is discussed in the manuscript. We suggest that this table be presented more concisely by displaying only relevant information.
- The outcome associated with the anti-hypertension discussion refers to table 2, but the explanation in the "Laboratory and radiology characteristics" section is not clear which table it relates to.

Page 10

- The proportional hazards assumption is critical for Cox regression to work correctly. One of the essential assumptions is that the risk ratio between any two persons remains constant over time. In Figure 3, we see the intersection of the Kaplan-Meier curves for the two groups being compared. As a result, Hazard ratio reporting in this group is less precise. We recommend that authors report RR rather than HR unless they have confirmed that the Cox regression assumptions have been satisfied.

Page 11

- "figure 3" - Make sure that all words are written in English.

Page 13

- The conclusion does not represent the results of the study. Please be consistent with your results to conform to a conclusion. The authors emphasize the dysregulation of the immune system, but the authors do not sufficiently explain the dysregulation of the immune system in the study's findings or discussions. We recommend that the writer leave this point out of the discussion and conclusion.

Is the work clearly and accurately presented and does it cite the current literature?

Yes

If applicable, is the statistical analysis and its interpretation appropriate?

Partly

Are all the source data underlying the results available to ensure full reproducibility?

Yes

Is the study design appropriate and is the work technically sound?

Partly

Are the conclusions drawn adequately supported by the results?

Partly

Are sufficient details of methods and analysis provided to allow replication by others?

Yes

Reviewer Expertise:

Cardiovascular, adipose tissue angiogenesis, cardiac hypertrophy, Senescence, Endothelin, Adipokine

We confirm that we have read this submission and believe that we have an appropriate level of expertise to confirm that it is of an acceptable scientific standard, however we have significant reservations, as outlined above.

# Response

**Published:** 2021-06-22

**Authors:** Satriyo Dwi Suryantoro

**Affiliations:** Departement of Internal Medicine, Faculty of Medicine, Airlangga University, Indonesia

Dear Dr Dhite

 Thank you for the helpful and considerate advices. We will revise soon.
